# DNA-PKcs is required for cGAS/STING-dependent viral DNA sensing in human cells

**DOI:** 10.1016/j.isci.2023.108760

**Published:** 2023-12-15

**Authors:** Dayana B. Hristova, Marisa Oliveira, Emma Wagner, Alan Melcher, Kevin J. Harrington, Alexandre Belot, Brian J. Ferguson

**Affiliations:** 1 Department of Pathology, University of Cambridge, Tennis Court Road, Cambridge CB2 1QP, UK; 2 The Institute of Cancer Research, London SW7 3RP, UK; 3 Centre International de Recherche en Infectiologie, Inserm, U1111, Université Claude Bernard, Lyon, France

**Keywords:** Virology, Molecular biology

## Abstract

To mount an efficient interferon response to virus infection, intracellular pattern recognition receptors (PRRs) sense viral nucleic acids and activate anti-viral gene transcription. The mechanisms by which intracellular DNA and DNA viruses are sensed are relevant not only to anti-viral innate immunity, but also to autoinflammation and anti-tumour immunity through the initiation of sterile inflammation by self-DNA recognition. The PRRs that directly sense and respond to viral or damaged self-DNA function by signaling to activate interferon regulatory factor (IRF)-dependent type one interferon (IFN-I) transcription. We and others have previously defined DNA-dependent protein kinase (DNA-PK) as an essential component of the DNA-dependent anti-viral innate immune system. Here, we show that DNA-PK is essential for cyclic GMP-AMP synthase (cGAS)- and stimulator of interferon genes (STING)-dependent IFN-I responses in human cells during stimulation with exogenous DNA and infection with DNA viruses.

## Highlights

• Human fibroblasts use the canonical cGAS/STING pathway for intracellular DNA sensing
• DNA-PKcs is required for the activation of this pathway in a kinase-independent manner
• DNA-PKcs KO cells do not activate STING in response to DNA or infection with DNA viruses
• SCID patients with the DNA-PKcs mutation L3062R have hyperactive STING signaling

## Graphical abstract

**Figure:**
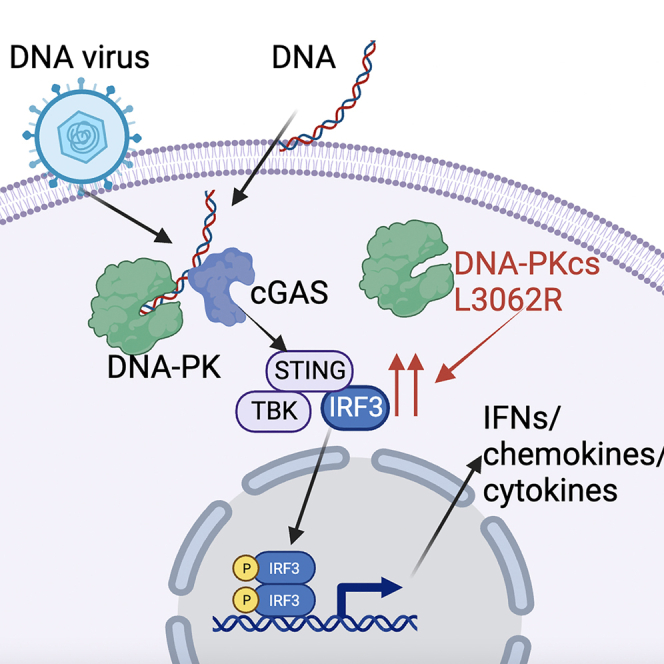

## Introduction

The ability of cells to sense and respond to pathogens by producing type I interferons (IFN-I) and inflammatory mediators is essential for host defense against infection. Pattern recognition receptors (PRRs) that bind nucleic acids and drive the transcription of IFN-I are specifically required to control virus infections.^1^ IFN-I acts in an autocrine or paracrine fashion to upregulate the expression of proteins that establish the anti-viral state in infected tissues, whilst the secretion of chemokines and cytokines attracts and activates tissue-resident and circulating leukocytes to help clear the infection.^2^

In the initial events of detection of viruses, genomic nucleic acids trigger the activation of PRRs that bind DNA or RNA directly and signal downstream to activate the transcription factors interferon regulatory 3 (IRF3) and nuclear factor kappa B (NF-κB). These active transcription factors move to the nucleus to initiate interferon and inflammatory gene activation. In the context of DNA virus infections, multiple receptors that activate this pathway have been identified. The DNA-binding PRRs cGAS, DNA-PK and interferon-inducible protein 16 (IFI16) function to sense and respond to DNA-containing pathogens and drive the innate and subsequent adaptive immune response to virus infection.^3^^,^^4^^,^^5^^,^^6^^,^^7^^,^^8^^,^^9^

The DNA-dependent protein kinase (DNA-PK) complex consists of a phosphatidylinositol 3-kinase-related protein kinase (PIKK) family catalytic subunit called DNA-PKcs, and two DNA-binding regulatory subunits Ku70 and Ku80. This heterotrimeric complex functions in non-homologous end-joining (NHEJ) that repairs nuclear double-stranded DNA breaks by directly binding broken ends of genomic DNA. We previously defined a function for DNA-PK in the sensing of intracellular DNA and DNA virus infections via the activation of an IRF3-dependent pathway.^4^ Others have re-affirmed the function of DNA-PK in sensing viruses and established DNA-PK as a critical regulator of anti-viral immunity.^7^^,^^8^^,^^9^^,^^10^^,^^11^^,^^12^ In order to activate IRF3, viral DNA-sensing PRRs signal via the activation of an adaptor protein, STING.^13^ STING binds the second messenger 2′-3′cGAMP, a product of the enzyme activity of cGAS^14^ and subsequently drives downstream IRF3 and NF-κB activation by a mechanism that requires the IKK family kinases including TANK-binding kinase-1 (TBK-1).^15^^,^^16^ DNA-PK has been reported to act in STING-dependent and -independent pathways depending on the cell type and activation context,^17^^,^^18^ and to activate IRF3 but not NF-κB-dependent signaling during infection or stimulation.^4^ DNA-PK is also itself a target of multiple viral counter-defence mechanisms encoded by large DNA viruses. Poxviruses and herpesviruses encode proteins that bind and/or target DNA-PK for degradation during infection,^19^^,^^20^^,^^21^ providing further evidence for the importance of this complex in the regulation of anti-viral immunity.

Here, we establish that DNA-PK functions in human cells to sense and respond to intracellular dsDNA and to the vaccinia virus (VACV) and herpes simplex virus 1 (HSV-1) viral DNA to drive interferon production. DNA-PK functions in the same pathway as cGAS and STING in this context and is required for STING activation and subsequent IRF3-dependent interferon and chemokine production. This work thereby establishes the function of DNA-PK in STING-dependent anti-viral immune responses in human cells.

## Results

### Human fibroblasts respond to intracellular DNA by activating STING/TBK1/IRF3

To establish a model system for studying the innate immune function of DNA-PK in human cells, we assessed multiple human cell lines for their ability to activate an interferon response to intracellular dsDNA. We found that, although many human cell lines did not respond to the transfection of dsDNAs, human foreskin fibroblasts (HFF) mounted a robust response. Fibroblasts are primary targets of virus infection and help coordinate multiple aspects of innate immunity and inflammation.^4^^,^^22^^,^^23^ Some redundancy exists in the reported DNA sensing pathways, with different sensors functioning in different contexts. There are, however, few reports dissecting the mechanisms that exist in specific human cell types to sense DNA and DNA virus infections. We therefore first assessed the existence of the canonical intracellular DNA sensing pathway in human fibroblasts (Figure S1). Following dsDNA transfection, HFFs activate STING, TBK1 and IRF3, as evidenced by the phosphorylation of these signaling pathway components (Figure S1A). Activation of this pathway leads to IFN-I and chemokine (CXCL10) transcription (Figure S1B), key target genes directly transcriptionally activated by DNA-dependent IRF3 activity,^4^ although we did not detect any induction of type III interferon or interferon alpha above background levels, and only very low levels of NF-κB-dependent transcripts, such as *NFKBIA* (Figure S1B).

### Intracellular DNA sensing in human fibroblasts requires cGAS, STING, and TBK1/IKKε

**Figure 1:**
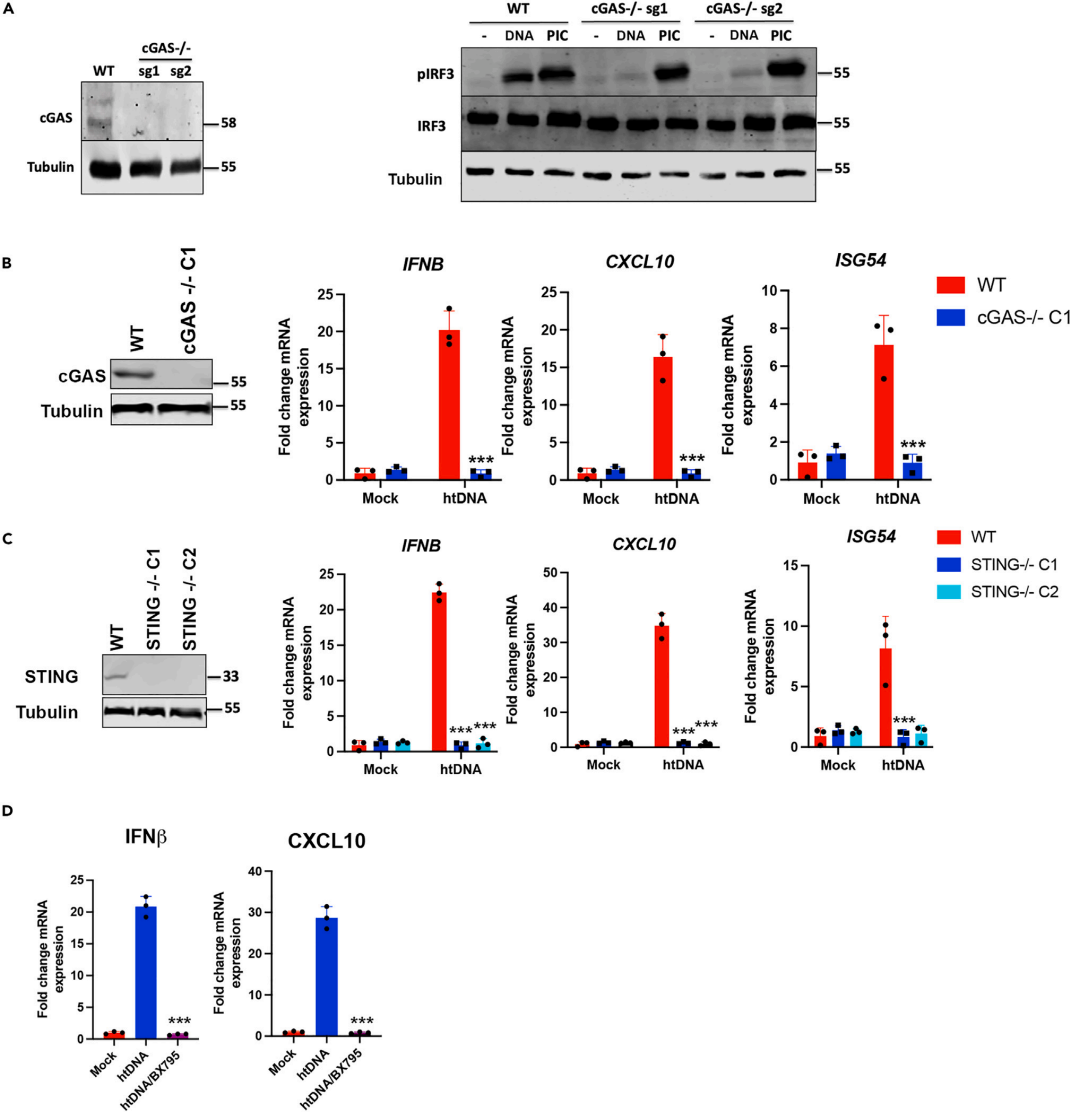
The interferon response to dsDNA stimulation in HFFs is dependent on cGAS, STING and TBK1 (A) cGAS knockout cell line pools were generated by CRISPR/Cas9 with two sgRNAs targeting different exons of the *MB21D1* gene (sg1 and sg2) and were immunoblotted with anti-cGAS antibody. WT (cGAS^+/+^) and cGAS^−/−^ cells were stimulated with stimulated with htDNA or poly(I:C) and analyzed by immunoblotting with the indicated antibodies. (B) A cGAS−/− clonal cell line (cGAS^−/−^ C1) was derived from cGAS−/− sg1 pool and immunoblotted with anti-cGAS antibody. WT (cGAS^+/+^) and cGAS^−/−^ C1 cells were stimulated with htDNA and analyzed by qRT-PCR 6 h later for the transcription of *IFNB*, *CXCL10* and *ISG54*. (C) STING knockout clonal cell lines (STING−/− C1 and C2) were generated by CRISPR/Cas9 using an gsRNA targeting the *TMEM173* gene and were immunoblotted with an anti-STING antibody. WT (STING^+/+^) and STING^−/−^ cells were stimulated with htDNA and analyzed by qRT-PCR 6 h later for the transcription of *IFNB* and *CXCL10* and *ISG54*. (D) WT HFFs were pre-treated with the TBK1/IKKε inhibitor BX795, stimulated by transfection with htDNA, and analyzed by qRT-PCR 6 h later for the transcription of *IFNB* and *CXCL10*. Data is representative of at least two biological repeats and presented as mean ± SEM. Data was analyzed by two-tailed Student’s T test with n = 3; ∗∗∗p < 0.001.

We next set out to define the pathway leading to IRF3 activation in response to DNA-induced stimulation in HFFs. To do this, we initially created cGAS knockout pools by CRISPR using guide RNAs (sg1 and sg2) that targeted two different exons in the human *MB21D1* gene and assessed their capacity to activate IRF3 in response to intracellular nucleic acid stimulation. WT cells were able to activate IRF3 in response to htDNA or RNA (poly(I:C)) stimulation but this activation was specifically lost in cGAS^−/−^ cells in response to DNA stimulation, indicating that cGAS is required for DNA-driven IRF3 activation in human fibroblasts (Figure 1A). As there was some residual IRF3 activation in CRISPR pools made with sgRNA2 we made a clonal cell line from the pool made with sgRNA1, (cGAS^−/−^ C1) and carried out further stimulation assays that showed undetectable DNA-driven interferon and chemokine transcription in cGAS^−/−^ cells (Figure 1B). In the same way we also made clonal cell lines lacking STING. Again, DNA-driven interferon and chemokine transcription was undetectable in STING^−/−^ cells (Figure 1C). Use of the TBK1/IKKε inhibitor, BX795, indicated that this DNA sensing pathway is also completely dependent on these kinases (Figure 1D). As such, we show here that the intracellular DNA sensing pathway in human fibroblasts uses the canonical cGAS/STING/TBK1/IRF3 signaling axis and that no detectable cGAS or STING-independent pathways function in this cell type.

### DNA-PKcs is required for stimulator of interferon genes activation and interferon-I production in response to DNA stimulation

**Figure 2:**
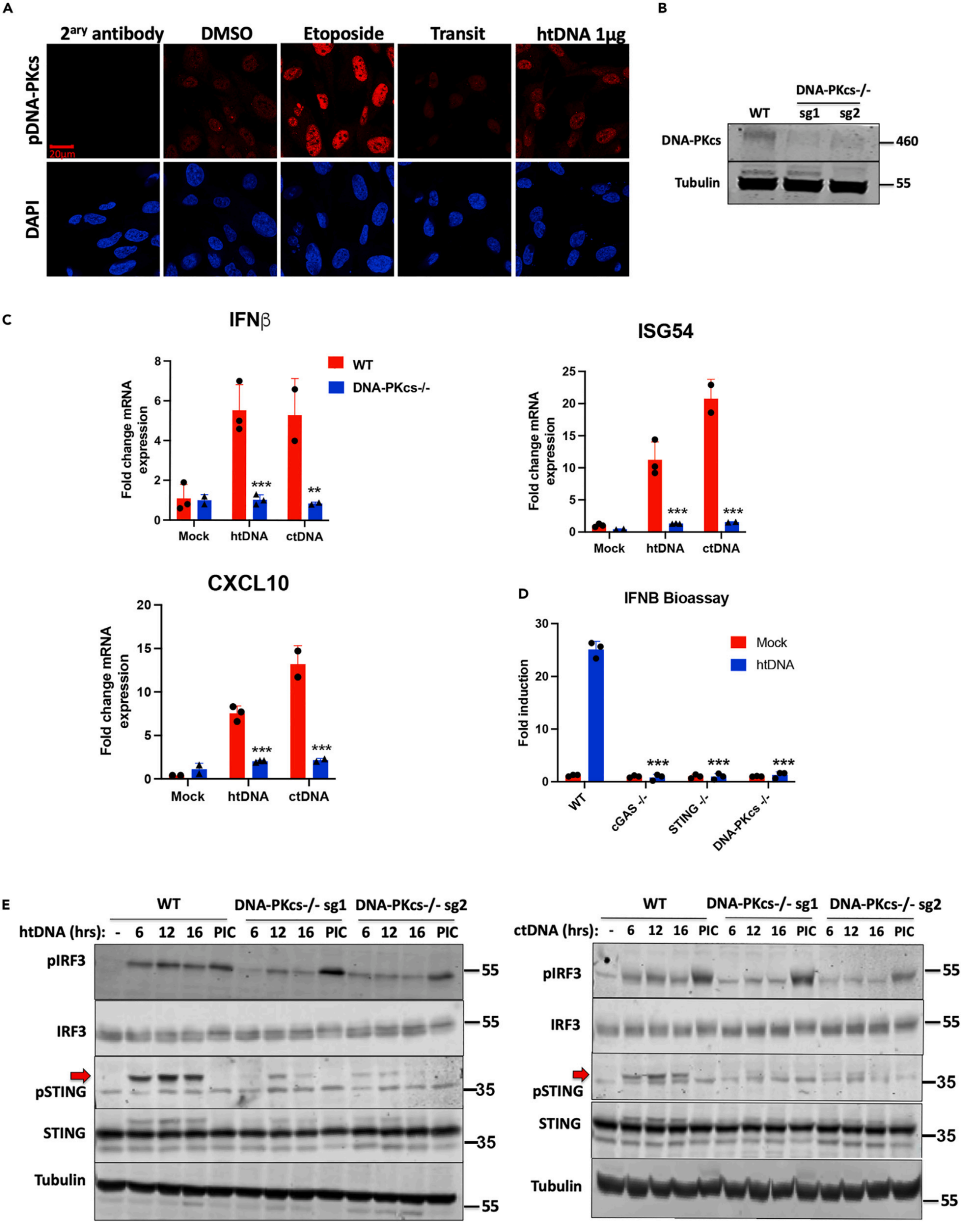
DNA-PKcs is required for STING-dependent sensing in human fibroblasts (A) DNA-PKcs is activated during intracellular DNA stimulation. Representative images of cells treated with etoposide, htDNA or carrier controls, fixed and stained by immunofluorescence with an antibody recognising phosphoserine 2056 on DNA-PKcs. Cells are counterstained with DAPI, scale bar = 20 μm. (B) DNA-PKcs knockout cell lines were generated by CRISPR/Cas9 with two guide RNAs targeting different exons of the *PRKDC* gene (sg1 and sg2) and were immunoblotted with anti-DNA-PKcs antibody. (C) WT (DNA-PKcs^+/+^) and DNA-PKcs^−/−^ cells were stimulated with htDNA or ctDNA and analyzed by qRT-PCR 6 h later for the transcription of *IFNB*, *ISG54* and *CXCL10*. (D) WT, DNA-PKcs^−/−^, STING^−/−^ or cGAS^−/−^ cells were stimulated with htDNA and IFNβ activity in the supernatant was measured 24 h later by bioassay. (E) WT (DNA-PKcs^+/+^) and DNA-PKcs^−/−^ cells were stimulated with ctDNA (top panel) or htDNA (bottom panel) or Poly(I:C) (PIC) for the indicated times and analyzed by immunoblotting with the indicated antibodies. Red arrows show the position of the phospho-STING specific band. Data is representative of at least two biological repeats and presented as mean ± SEM. Data was analyzed by two-tailed Student’s T test with n = 3; ∗∗p < 0.01, ∗∗∗p < 0.001.

To assess the role of DNA-PK in DNA sensing in human cells, we stimulated HFFs with htDNA and monitored activation of DNA-PKcs using an autophosphorylation-specific antibody that recognises pS2056. Stimulation of cells with DNA or using etoposide to initiate DNA damage resulted in an increase in pS2056 signal above background levels (Figure 2A) indicating the activation of DNA-PK, a process that usually occurs via DNA end binding. We next generated two CRISPR KO HFF lines using sgRNAs targeting different DNA-PKcs exons (Figure 2B). Stimulation of these DNA-PKcs KO human fibroblasts resulted in complete loss of *IFNB* and *CXCL10* transcription (Figure 2C) and, also, reduction in the transcription of the IRF3-specific target gene *ISG54* (Figure 2C). IFN protein secretion was also analyzed using an IFN-I bioassay^24^ in WT, DNA-PKcs^−/−^, STING^−/−^ and cGAS^−/−^ cells and confirmed that loss of any of these three individual components abrogates DNA-driven interferon production (Figure 2D). Analysis of intracellular signaling pathway activation in these cells indicated that DNA-PKcs loss resulted in significant reductions in DNA-driven STING and IRF3 activation, although intracellular RNA-driven IRF3 activation was unaffected (Figure 2E). As such, these data indicate that DNA-PKcs is essential for the cGAS/STING pathway of DNA sensing in human fibroblasts.

**Figure 3:**
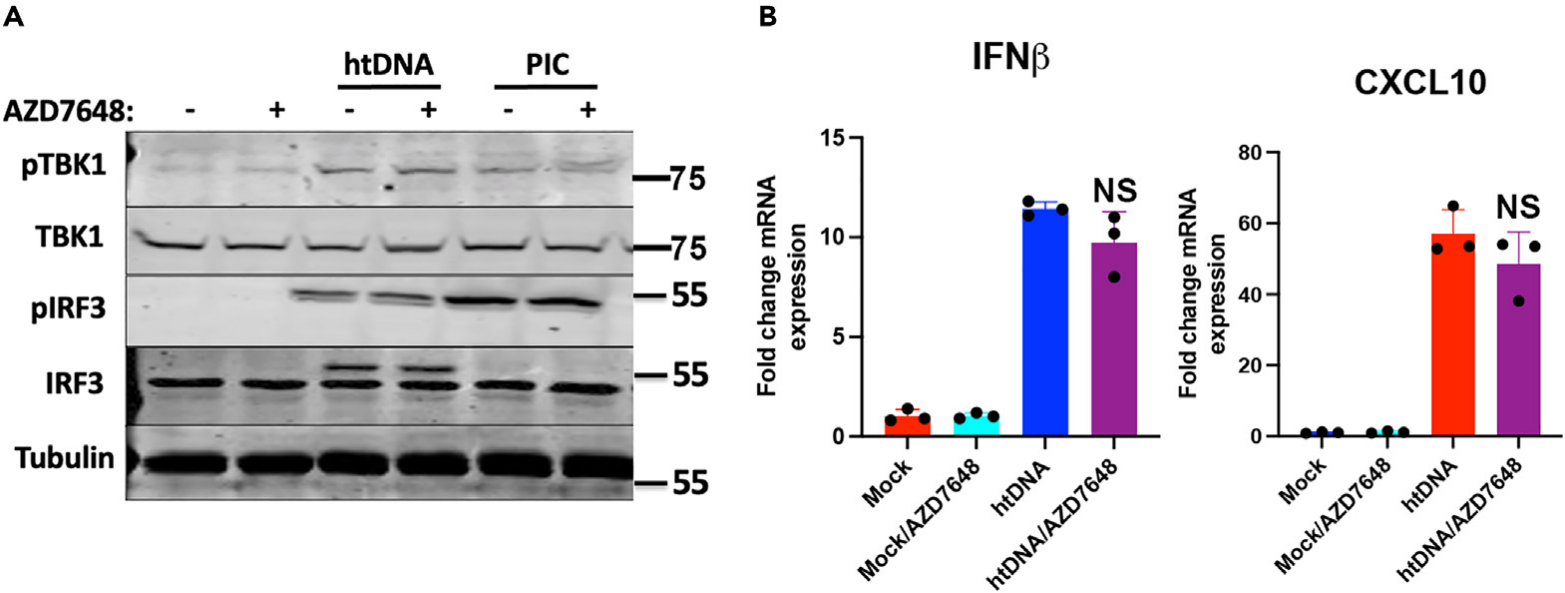
DNA-PKcs kinase activity is dispensable for DNA sensing Cells were pre-treated with AZD7648 for 1 h prior to transfection with htDNA or poly(I:C) and (A) immunoblotted for the indicated antibodies or (B) analyzed by qRT-PCR for *IFNB* and *CXCL10* 6 h post stimulation. Data is representative of at least two biological repeats and presented as mean ± SEM. Data was analyzed by two-tailed Student’s T test with n = 3; NS = non-significant.

The kinase activity of DNA-PKcs is required for multiple aspects of DNA-PK function, including its roles in DNA damage repair and in V(D)J recombination. Here, we asked whether the inhibition of the DNA-PKcs kinase domain with small molecule inhibitors could impact its function in DNA sensing. We first used the inhibitor NU7441 that can inhibit DNA-PKcs activity (Figure S2A) and found that the pre-treatment of cells with NU7441 enhanced the DNA-driven interferon responses (Figure S2B). As this result was incongruous with our data from DNA-PKcs KO cells, we hypothesised that this compound may have an off-target effect that impacts this signaling pathway. Indeed, we found that NU7441 could activate TBK1 in the absence of DNA-PKcs (Figure S2C), indicating a strong off-target effect on the DNA sensing pathway of this molecule and, hence, that it is unsuitable in this context. As an alternative, we used the more-specific compound AZD7648.^25^ This compound can inhibit DNA damage-dependent histone phosphorylation (γH2AX) (Figure S3), a process that is DNA-PKcs-dependent, but had no impact on DNA-dependent TBK1 and IRF3 phosphorylation (Figure 3A), or *IFNB* transcription (Figure 3B) in HFFs. As such, the kinase activity of DNA-PKcs was not found to be required in the context of DNA sensing in human fibroblasts.

### DNA-PKcs is essential for the stimulator of interferon genes-dependent sensing of DNA viruses

**Figure 4:**
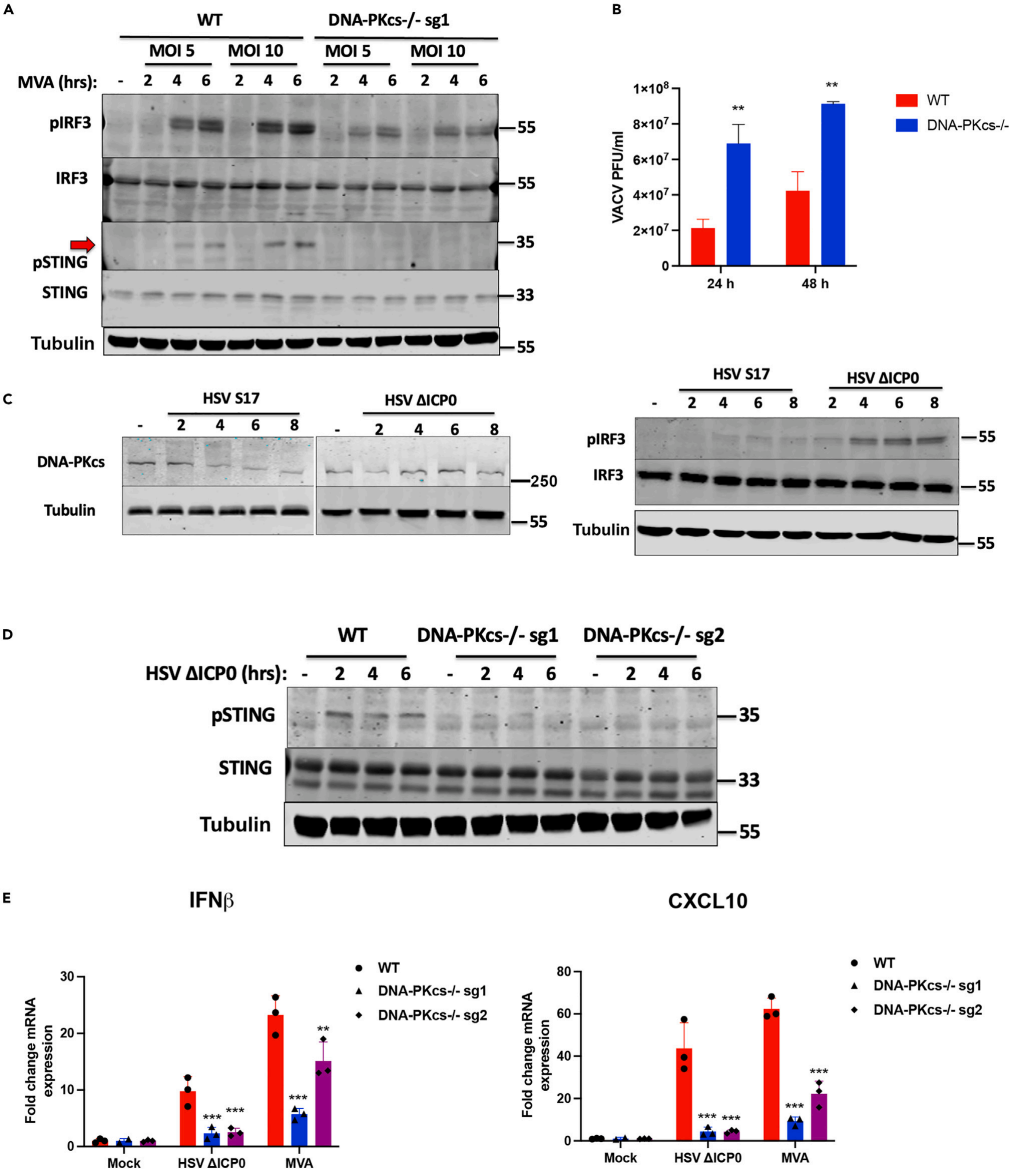
DNA-PKcs is required for the innate sensing of DNA viruses in human cells (A) WT (DNA-PKcs^+/+^) and DNA-PKcs^−/−^ cells were infected with MVA at the indicated MOIs and analyzed by immunoblotting with the indicated antibodies. Red arrow shows the position of the phospho-STING specific band. (B) WT (DNA-PKcs^+/+^) and DNA-PKcs^−/−^ cells were infected with VACV TBio 6517 at MOI 0.01 and the production of infectious virions was quantified 24 or 48 h later by plaque assay on BSC-1 cells. (C) HFFs were infected with WT (S17) or *dl1043* HSV-1 (ΔICP0) at MOI 5 and immunoblotted with the indicated antibodies. (D) WT (DNA-PKcs^+/+^) and DNA-PKcs^−/−^ cells were infected with *dl1043* HSV-1 (ΔICP0) at MOI 5 and immunoblotted with the indicated antibodies). (E) WT (DNA-PKcs^+/+^) and DNA-PKcs^−/−^ cells were infected with MVA or *dl1043* HSV-1 (ΔICP0) at the indicated times and analyzed by qRT-PCR for *IFNB* and *CXCL10* 6 h post infection. Data is representative of at least two biological repeats and presented as mean ± SEM. Data was analyzed by two-tailed Student’s T test with n = 3; ∗∗p < 0.01, ∗∗∗p < 0.001.

To determine the contribution of DNA-PKcs to the innate sensing of DNA viruses, we used two infection models with dsDNA viruses. Modified vaccinia Ankara (MVA) is a derivative of the vaccinia virus strain chorioallantois vaccinia Ankara (CVA) produced by extensive passage in chicken cells. MVA has lost numerous immunomodulatory proteins as well as the ability to replicate in human cells. MVA can still enter most human cells, however, and presents large amounts of viral DNA into the cytoplasm that can activate cGAS/STING signaling.^4^^,^^26^^,^^27^ Infection of HFFs with MVA resulted in STING and IRF3 phosphorylation that was markedly reduced in the absence of DNA-PKcs (Figure 4A), indicating that DNA-PKcs is required for vaccinia virus-driven activation of the STING/TBK1/IRF3 signaling axis. As MVA is non-replicative in human cells, we used a replicating strain of VACV (TBio 6517) to analyze the impact of DNA-PKcs loss on the yield of virus from infected cells. In a multi-step growth curve analysis, we found that cells lacking DNA-PKcs produced a significantly enhanced virus yield (Figure 4B), consistent with its role in host defense against DNA virus infections.

Next, we used herpes simplex virus 1 (HSV-1) strain 17, which we found not to activate IRF3 during HFF infection, but that did rapidly degrade DNA-PKcs (Figure 4C), as previously reported in other cell lines.^21^ HSV-1 encodes an immediate-early expressed protein, infected cell polypeptide 0 (ICP0), that has E3 ubiquitin ligase activity. ICP0 is responsible for the degradation of DNA-PKcs^28^ and other targets implicated in anti-viral immunity.^29^^,^^30^^,^^31^^,^^32^ We hypothesised that this degradation of DNA-PKcs by ICP0 may be interfering with the ability of the host cell to sense viral DNA and, therefore, we used the HSV-1 mutant *dl1043* virus that lacks the expression of ICP0. Unlike strain 17, *dl1043* infection of HFFs did not result in a reduction of DNA-PKcs expression but did result in measurable IRF3 activation (Figure 4C). Consistent with MVA infection, following *dl1043* infection wild-type HFFs could activate STING, but this was absent in DNA-PKcs KO cells (Figure 4D). In the absence of DNA-PKcs, MVA and *dl1043-*driven *IFNB* and *CXCL10* transcription were both significantly reduced compared with the infection of wild-type cells (Figure 4E). These data show that DNA-PKcs is required for the IFN-I response to *poxvirus* and *herpesvirus* infections consistent with its essential function in the anti-viral DNA sensing pathway in human fibroblasts.

### The human mutation DNA-PKcs L3062R is a gain of function mutation for intracellular DNA sensing

**Figure 5:**
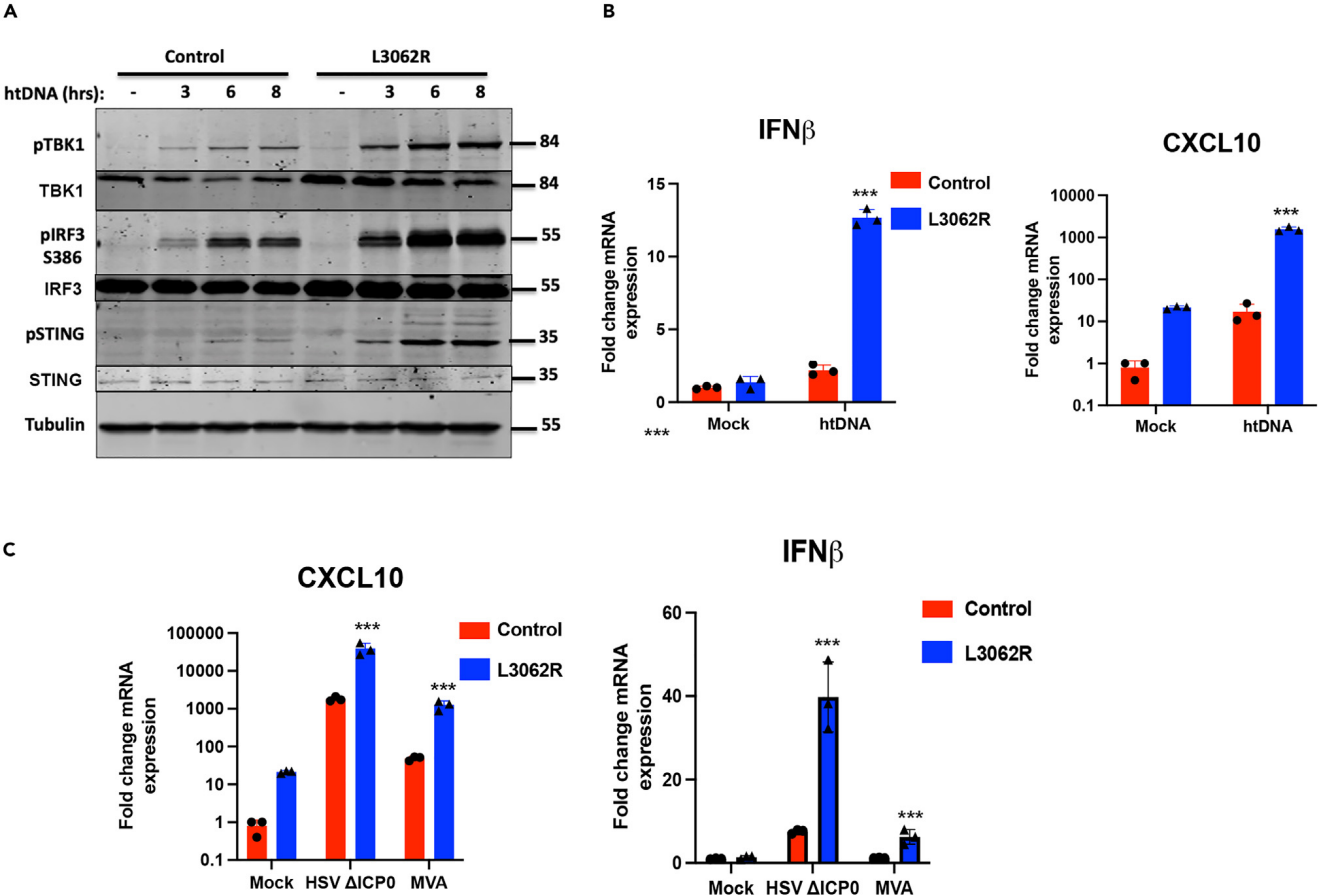
The human DNA-PKcs L3062 mutation enhances STING signaling during DNA stimulation and DNA virus infection (A) Primary skin fibroblasts from healthy donor or a patient harboring the DNA-PKcs L3062 mutation were stimulated with HT-DNA and (A) immunoblotted with the indicated antibodies or (B) analyzed by qRT-PCR for *IFNB* and *CXCL10* 6 h post stimulation. (C) Primary skin fibroblasts from healthy donor or a patient harboring the DNA-PKcs L3062 mutation were infected with MVA or HSV-1ΔICP0 at MOI 5 and analyzed by qRT-PCR for *IFNB* and *CXCL10* 6 h post infection. Data is representative of at least two biological repeats and presented as mean ± SEM. Data was analyzed by two-tailed Student’s T test with n = 3; ∗∗∗p < 0.001.

Loss of DNA-PKcs function is associated with severe-combined immunodeficiency disorder (SCID), characterised by loss of B and T cell repertoires due to a lack of V(D)J recombination. Since the discovery that DNA-PKcs is essential for V(D)J recombination in mice, several human polymorphisms have been discovered that result in a similar primary immune deficiency. Once such rare mutation is L3062R.^33^ This mutation results in SCID in humans and is associated with a loss of the DNA repair function of DNA-PKcs, resulting in failed V(D)J recombination. The amino acid residue L3062 lies on the surface of DNA-PKcs in a region of the protein associated with Artemis-binding and does not impact the kinase activity. Of interest is the observation that patients carrying the biallelic L3062R DNA-PKcs variant exhibit interferon signatures in the blood.^34^ Interestingly, this positive signature persists three years after the bone marrow transplantation in one patient (IFN signature >16, normal value < 2.3). We analyzed the ability of primary skin fibroblasts from healthy donor or a patient carrying this mutation for their response to htDNA stimulation. Cells carrying the L3062R mutation showed enhanced STING/TBK1/IRF3 activation and *IFNB* transcription in response to htDNA stimulation (Figures 5A and 5B). Cells carrying this variant also showed enhanced CXCL10 transcription when resting, indicating increased basal inflammatory signaling (Figure 5B). In cells harboring the L3062R mutation, *IFNB* and *CXCl10* transcription were also enhanced in MVA and HSV-infected cells, consistent with this mutant having enhanced function in the context of STING-dependent signaling (Figure 5C).

The dual function of DNA-PKcs in dsDNA break repair and innate immune sensing of intracellular DNA likely requires DNA-PKcs to activate different downstream signaling pathways. Although the L3062R mutation disrupts the DNA repair function of DNA-PKcs by perturbing its interaction with Artemis, our data here suggests that this same mutation can enhance the activity of DNA-PK/cGAS/STING signaling pathway, potentially by enhancing interactions with other proteins in this pathway. These data provide further direct evidence of the function of DNA-PKcs in the regulation of intracellular DNA sensing in humans.

## Discussion

The function of DNA-PK in the innate sensing of DNA and DNA viruses has been described in several contexts. In mice and in murine fibroblasts, DNA-PKcs, Ku70 and Ku80 are required for the intracellular DNA-driven IRF3 activation and for sensing vaccinia virus and HSV-1.^4^ In monocytic THP-1 cells, Ku70 and Ku80 are required for sensing HSV-1 by triggering cGAS activation.^8^ In HeLa cells, Ku70 interacts with the retrovirus human T cell leukemia virus-1 (HTLV-1) reverse transcription intermediate DNAs to drive IRF3-dependent interferon beta responses.^12^ In human endothelial cell lines, DNA-PK has been reported to regulate the interferon response to the herpesvirus KSHV^11^ and Ku70 is reported to regulate the chemokine response to hepatitis B virus (HBV) infection.^35^ Our data here confirm the importance of DNA-PKcs in the sensing of both cytoplasmic and nuclear-replicating DNA viruses and its role in initiating the generation of a type-I interferon response to the infection of human cells.

There is increasing evidence that the DNA-PK complex regulates one or more pathways downstream of intracellular dsDNA detection. In both murine and human fibroblasts, DNA-PKcs is required for STING-dependent IRF3 activation that leads to the generation of a classical anti-viral type-I interferon response. In this study, we show that human fibroblasts lacking DNA-PKcs fail to activate STING/IRF3 signaling and subsequent gene transcription in response to intracellular DNA stimulation and DNA virus infection. There are reports of a similar role of the Ku proteins in monocytic cells^8^ and of a potentially separate pathway that links Ku with IFN-III production, but that also requires STING.^10^^,^^17^ In parallel, there is a report of DNA-PK activating an STING-independent pathway to IRF3 activation in a human leukemia cell line.^18^ The mechanisms underlying these co-operative or independent pathways are likely to involve direct interplay between DNA-PKcs/Ku70/Ku80 and other DNA sensing PRRs. Indeed, others^8^^,^^36^ have shown that Ku and/or DNA-PKcs can interact with cGAS and there is a reported interaction between DNA-PK and another DNA sensing PRR, IFI16, that regulates the interferon response to HSV-1.^7^

Our data here, and in a previous report,^4^ indicate that the kinase activity of DNA-PK is not required for its ability to activate the STING/TBK1/IRF3 signaling axis. Use of cells from SCID mice that have a deletion in the last 82 amino acids of DNA-PKcs that inactivates the kinase domain,^4^ or use of specific DNA-PKcs kinase inhibitors consistently show that there is no effect of blocking the kinase activity on DNA-driven signaling. As such, it is likely that the role of DNA-PKcs/Ku70/Ku80 in innate sensing is a structural one, possibly in sensing specific structures or sequences of DNA, such as DNA ends^18^ or nicks and gaps in DNA viral genomes in concert with IFI16^7^^,^^37^ and delivering them to cGAS to drive STING activation. In this context, it maybe that DNA-PK can positively or negatively regulate DNA sensing, depending on cell type and context of the infection.^36^ Future studies will require more detailed structural and biochemical analyses of these processes and cell type-specific analyses using cells that are relevant to the infection processes being studied.

Further insight into the mechanisms by which DNA PRRs function in the context of infection can be obtained by studying the role of specific viral proteins that inhibit these host sensing pathways. During HSV-1 infection, DNA-PKcs is rapidly degraded in a manner dependent on ICP0 (Figure 4C),^21^ although the Ku proteins remain stable, consistent with the concept that all three DNA-PK components are required for DNA sensing. ICP0 can also target other proteins in the DNA sensing pathway, including IRF3 and IFI16,^29^^,^^30^ making it a multifunctional protein in the evasion of anti-viral host responses. Poxviruses encode the C16/C4 family of immunomodulatory proteins that use steric hindrance to block viral DNA from binding to the Ku proteins,^19^^,^^20^ resulting in a reduction of the DNA-driven interferon response. This mechanism is consistent with a DNA-binding, structural function of DNA-PK in the context of intracellular DNA sensing. Notably, many other DNA viruses express proteins that bind or interfere with the function of DNA-PK,^38^ although the function of many of these proteins in innate sensing is not yet described. This virus/host co-evolution can explain much of the complexity in these key anti-viral host responses that regulate DNA sensing and DNA-virus driven interferon responses.

Equally these same anti-viral DNA sensing systems can drive autoinflammatory and autoimmune responses to mis-localised or damaged self-DNA. The accumulation of damaged nuclear or mitochondrional DNA or undigested phagocytosed apoptotic bodies can result in STING-driven interferon and inflammatory cytokine production.^39^ Since DNA-PK is one of the multiple components of the anti-viral DNA sensing machinery that share functions in the host DNA damage response it may have been expected that the DNA-PKcs KO in HFFs would lead to the accumulation of DNA damage and subsequent background autoinflammation. We did not observe this, however, which could be due to redundancy in DNA damage response machineries compensating for loss of DNA-PKcs, or due to a role of DNA-PKcs in the activation of STING in sterile inflammation as well as in anti-viral responses, although this needs further investigation.

The discovery of patients with mutations in DNA-PKcs has provided further evidence for the function of this protein in innate immunity. The L3062R mutation analyzed here causes severe combined immunodeficiency (SCID) by disrupting V(D)J recombination and lymphocyte maturation. Here, we show that primary fibroblasts from patients with this mutation hyperactivate STING following DNA stimulation, by an unknown mechanism. This mutation is on the surface of DNA-PKcs in the region of the protein that is responsible for binding Artemis.^40^ It is hypothesised that this mutation disrupts DNA-PKcs/Artemis interactions and hence reduces the efficiency of double-strand DNA break repair and V(D)J combination. It is possible, but as yet unexplored, that this DNA-PKcs/Artemis interaction may also regulate DNA sensing, in particular as Artemis has been implicated in the manipulation of the terminal ends of viral genomes.^41^ This data also hints to a contribution of DNA-PK-dependent DNA sensing in sterile inflammation, as discussed above. The dysregulation of intracellular nucleic acid sensing contributes to multiple autoimmune and autoinflammatory diseases, such as Lupus and Aicardi-Goutieres syndrome.^39^^,^^42^ It is possible therefore, that DNA-PKcs contributes to the pathogenesis of such disorders and, in a similar manner, to the triggering of anti-tumour immune responses.^43^

### Limitations of study

The main limitations of this study are that much of the work is carried out in an immortalised cell line, that might not completely mimic innate immune responses in primary cells or *in vivo*. In addition, the data generated from this single cell type (fibroblasts) may not be broadly applicable across all cell types where different pathways might function.

## STAR★Methods

### Key resources table

**Table:** 

REAGENT or RESOURCE	SOURCE	IDENTIFIER
**Antibodies**		

anti-cGAS	Santa Cruz Biotech	Cat# sc-515777; RRID: AB_2734736
anti-DNA-PKcs	Thermo Fisher Scientific	Cat# MS-423-P1; RRID: AB_61152
anti-IFI16	Santa Cruz Biotech	Cat# sc-8023; RRID: AB_627775
anti-IkBa	Santa Cruz Biotech	Cat# sc-1643; RRID: AB_627772
anti-IRF3	Abcam	Cat# ab68481; RRID: AB_11155653
anti-Ku70	Abcam	Cat# ab3114; RRID: AB_2219041
anti-TBK1	Abcam	Cat# ab40676; RRID: AB_776632
anti-STING	CST	Cat# 13647; RRID: AB_2732796
anti-Tubulin	Millipore	Cat# 05-829; RRID: AB_310035
anti-HSV-1-ICP0	Santa Cruz Biotech	Cat# sc-53070; RRID: AB_673704
anti-pIRF3 (Ser386)	Abcam	Cat# ab76493; RRID: AB_1523836
anti-pTBK1 (Ser172)	CST	Cat# 5483S; RRID: AB_10693472
anti-pSTING (Ser366)	CST	85735
anti-γH2AX	Millipore	Cat# 05-636; RRID: AB_309864
Anti-mouse IgG 800nm	Li-Cor	Cat# 926-32210; RRID: AB_621842
Anti-rabbit IgG 680nm	Li-Cor	Cat# 926-68071; RRID: AB_10956166
Donkey anti-mouse IgG AF488	Thermo Fisher Scientific	Cat# A-21202; RRID: AB_141607
Donkey anti-mouse IgG AF546	Thermo Fisher Scientific	Cat# A10036; RRID: AB_2757557
Donkey anti-rabbit IgG AF546	Thermo Fisher Scientific	Cat# A10040; RRID: AB_2757562

**Bacterial and virus strains**		

MVA	Professor Geoffrey Smith	
HSV-1 *dL1043*	Professor Gill Elliot	
TBio 6517	Turnstone Biologics	

**Biological samples**		

Fibroblast from patients carrying biallelic p.Leu3062Arg mutation	CRB Biotech, Lyon, France	

**Chemicals, peptides, and recombinant proteins**		

herring testis DNA	Sigma	D6898
calf thymus DNA	Sigma	D3664
HMW Poly(I:C)	Invivogen	tlrl-pic
TransIT-LT1	Mirus Bio	MIR 2300

**Experimental models: Cell lines**		

Human foreskin fibroblasts	Professor Mike Weekes	
HEK293 clone 3C11	Professor Jan Rehwinkel	
Chicken embryonic fibroblasts	Pirbright Institute	
U20S	ECACC	92022711
BSC-1	Professor Geoffrey Smith	
BHK	Professor Geoffrey Smith	

**Oligonucleotides**		

qPCR primers	See Table S2	
sgRNAs	See Table S1	

### Resource availability

#### Lead contact

Further information and requests for resources and reagents should be directed to and will be fulfilled by the lead contact, Brian Ferguson (bf234@cam.ac.uk).

#### Materials availability

All unique reagents generated in this study are available from the lead contact with a completed materials transfer agreement.

#### Data and code availability

All data reported in this paper will be shared by the lead contact upon request.

This paper does not report original code.

Any additional information required to reanalyse the data reported in this paper is available from the lead contact upon request.

### Experimental model and study participant details

Fibroblast from patients carrying biallelic p.Leu3062Arg mutation were provided by the CRB Biotech, Lyon, France. The study was approved by the Medical Ethics Committee of Sud Est III (Lyon, France) and carried out in accordance with the Declaration of Helsinki principles. Patient provided written informed consent for inclusion of their details and samples in the study.

### Method details

#### Cells

Human immortalised foreskin fibroblasts (HFF-hTert), U20S, BSC-1, baby hamster kidney (BHK), HEK293 clone 3C11 (a kind gift from Jan Rehwinkel, Oxford University), and primary skin fibroblasts were cultured in DMEM with 10% v/v fetal calf serum (FCS) and 50 μg/mL Pen-strep. Chicken embryonic fibroblasts (CEF) were cultured in DMEM-F12 with Glutamax (Gibco), 5% v/v FBS, and 50 μg/mL pen-strep.

#### Viruses

Modified vaccinia Ankara (MVA) was grown on BHK cells and titrated on primary CEFs. Titrations were counted by immunostaining using an anti-VACV Lister cocktail antibody (RayBiotech, MD-14-1041), secondary anti-rabbit horseradish peroxidase (HRP)-conjugated antibody (Sigma, A6154) and True-blue substrate (KPL) to visualise plaques. VACV strain TBio 6517, currently in early clinical testing (NCT04301011), kindly provided by Turnstone Biologics, was grown and titrated on BSC-1 cells. For growth curve analysis, HFF-hTERT cells were infected with VACV TBio 6517 at multiplicity of infection (MOI) 0.01. 24 or 48 h later, cell lysates were prepared, frozen and thawed three times and sonicated to obtain cell-associated virus. Numbers of infectious virions were quantified by titration on BSC-1 cells. HSV-1 strains S17 (wild type) and *dl403* lacking the *ICP0* gene (HSV-1ΔICP0)^44^ were grown and titrated on U2OS cells and plaques were counted using toluidine blue staining.

#### Knockout cell line generation

HFF-hTERT-Cas9 cells were transduced with lentiviruses expressing gRNAs targeting *PRKDC*, *TMEM173* or *MB21D1* (Table S1). Pooled or clonal cell lines were selected with puromycin and analyzed by immunoblotting for successful editing.

#### Stimulations

HFF were seeded in tissue culture plates and, the following day, cells were transfected using TransIT-LT1 (Mirus Bio, USA) with herring testis (HT)-DNA, calf thymus (CT)-DNA, or Poly(I:C) and harvested 6 or 16 h post-transfection.

#### qRT-PCR

Cells were lysed in 250 μL of lysis buffer containing 4 M guanidine thiocyanate, 25 mM Tris pH 7, and 143 mM 2-mercaptoethanol and purified on silica columns (Epoch Life Science). Using 500 ng of RNA, cDNA was produced using SuperScript III reverse transcriptase, following the manufacturer’s protocol (Thermo Scientific, Waltham, MA, USA). cDNA was used for quantitative PCR (qPCR) in a final volume of 10 μL qPCR was performed using SybrGreen Hi-Rox (PCR Biosystems Inc.) using primers described in Table S2. Fold change in mRNA expression was calculated by relative quantification using GAPDH as endogenous control.

#### Interferon bioassay

Was performed as previously described.^24^ Briefly, supernatant from HFFs stimulated with htDNA was incubated with HEK293 cells expressing firefly luciferase under control of the IFNβ promoter, and luciferase activity was measured after 24 h luciferase using luciferin substrate.

#### Immunoblotting

Cells were lysed in radioimmunoprecipitation assay (RIPA) lysis buffer (50 mM Tris-HCL pH8, 150mM NaCl, 1% NP-40, 0.1% SDS, 0.5% Na Deoxycholate), cOmplete Mini EDTA-free protease inhibitors (Roche), as well as PhosSTOP phosphatase inhibitor cocktail (Roche) and quantified using the bicinchoninic (BCA) assay (Thermo Scientific). Protein samples were run on 10–12% SDS polyacrylamide gels on a Bio-Rad Protean III system or 4–12% bis-Tris gradient Nu-PAGE gels on a Novex Mini-Cell system (Invitrogen) (Invitrogen). After electrophoresis, proteins were transferred onto nitrocellulose membrane, immunoblotted with the indicated antibodies (key resources table) and imaged by a Li-Cor Odyssey CLx.

#### Immunofluorescence microscopy

Cells were seeded in a 24-well plate on sterile 13 mm coverslips and fixed for 10 min with cold 4% w/v formaldehyde (Fisher Scientific) and cold 8% w/v formaldehyde in HEPES buffer. Cells were permeabilised for 5–10 min with 0.25% v/v Triton X-100 in PBS, blocked with 5% w/v milk in PBS at RT for 1 h. The cells were then incubated overnight at 4°C with primary antibody (key resources table) at the indicated dilution in 1% w/v milk in PBS. Following this, the cells were washed with PBS three times and were incubated at RT for 30 min in the dark with secondary antibody diluted 1:1000 in 1% w/v milk in PBS. Coverslips were mounted onto slides with 10 μL of mounting solution (25% glycerol v/v, 0.1 M Tris pH 8.5, 10% Mowiol 4–88 w/v containing 4′, 6-diamidino-2- phenylindole (DAPI). A Zeiss Pascal Confocal microscope was used to visualise the samples.

### Quantification and statistical analysis

Three independent experimental replicates were performed for all experiments unless otherwise stated. The applied statistical tests are indicated in the figure legends, where n = number of independent biological repeats. For all tests, p values less than or equal to 0.05 were deemed significant. All statistical analyses were performed using GraphPad Prism version 10 for Mac OS X (GraphPad software, USA).
