# Supplementary material for: DNA-PKcs is required for cGAS/STING-dependent viral DNA sensing in human cells
Source: iScience. 2023 Dec 15;27(1):108760. doi: 10.1016/j.isci.2023.108760 (PMC10805666; doi:10.1016/j.isci.2023.108760)
Supplement: Document S1. Figures S1–S3 and Tables S1 and S2 [file mmc1.pdf]

iScience, Volume *27*

Supplemental information

DNA-PKcs is required for cGAS/STING-dependent viral DNA sensing in human cells

Dayana B. Hristova, Marisa Oliveira, Emma Wagner, Alan Melcher, Kevin J. Harrington, Alexandre Belot, and Brian J. Ferguson

Supplementary Data

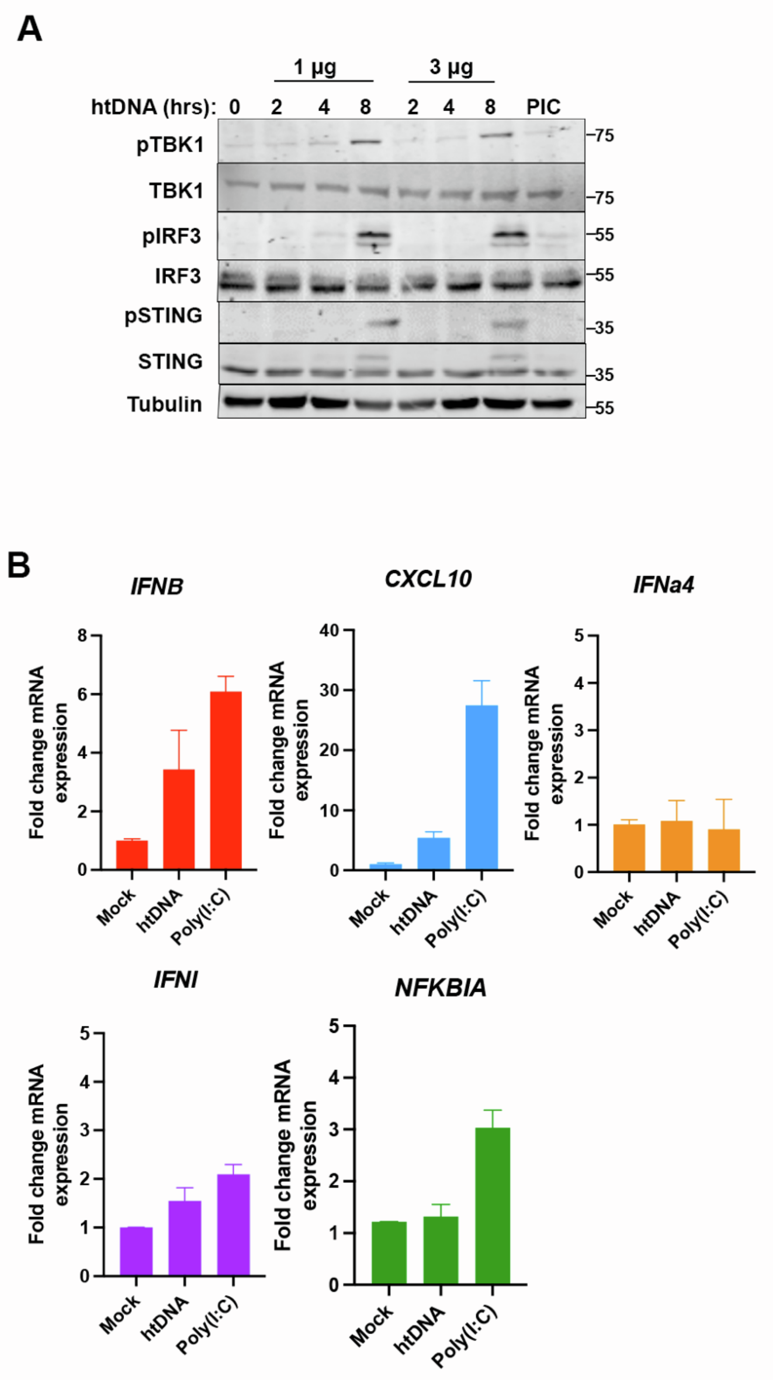

Supplementary Figure S1. Human fibroblasts respond to intracellular DNA stimulation by activating the STING/IRF3/TBK1 signalling axis, related to Figure 1. A) HFFs were stimulated by transfection with 1 or 3 µg/ml of htDNA for the indicated times and immunoblotted for

the indicated proteins. B) HFFs were stimulated by transfection with 3 μg/ml htDNA or 1 μg/ml poly(I:C) and analysed by qRT-PCR 6 h later for the indicated genes. n=3 *p<0.5, **p<0.01.

Supplementary Figure S2

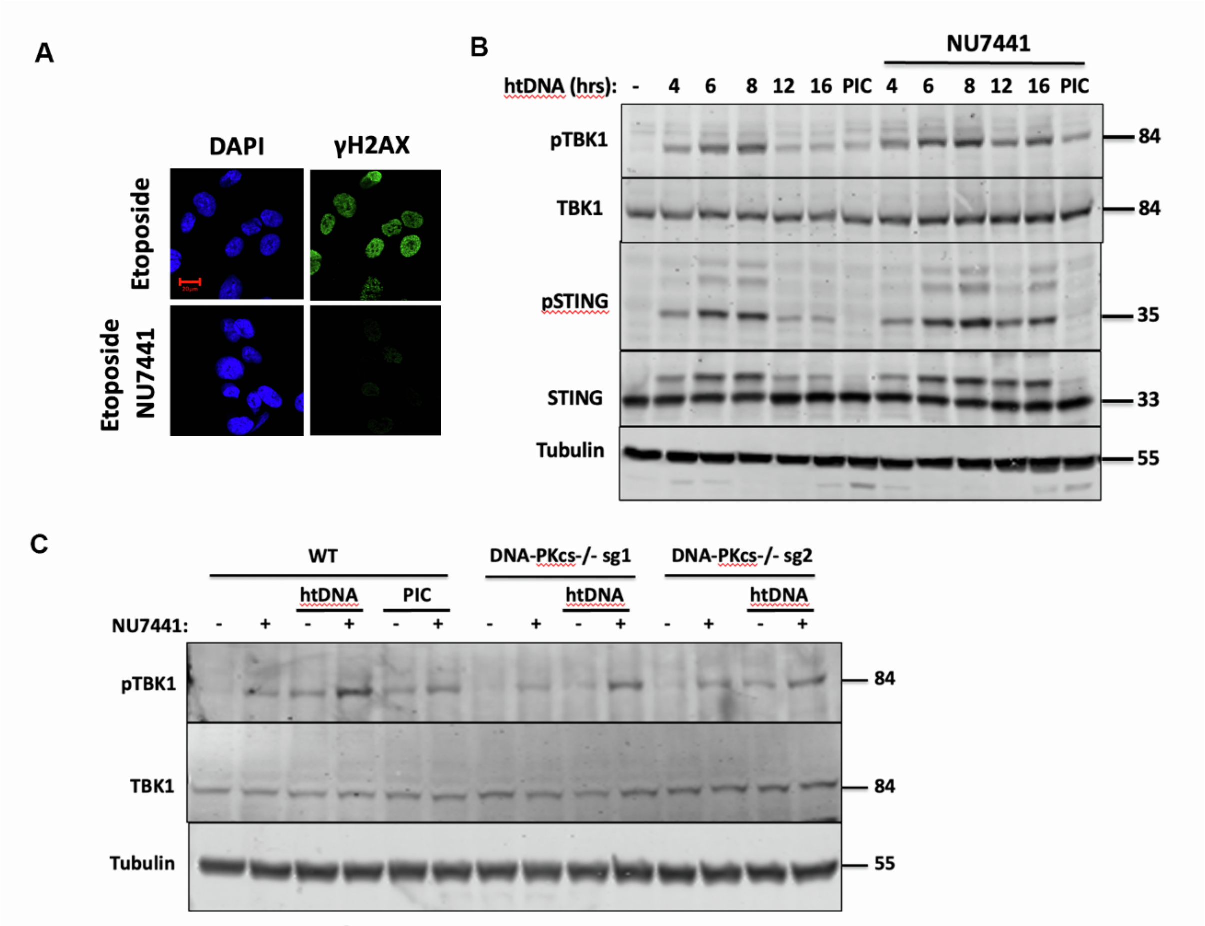

Supplementary Figure S2. Nu7441 enhances DNA sensing by activating TBK1 independently of DNA-PKcs inhibition, related to Figure 3. A) HFFs were pre-treated with 2 µg/ml Nu7441 or carrier control and then with 30 µM etoposide for 2 h before being fixed and stained for anti-γH2AX. Scale bar=20 µm B) HFFs were pre-treated with 2 µg/ml Nu7441 or carrier control and stimulated with htDNA for the indicated times and immunoblotted with the indicated antibodies. C) WT (DNA-PKcs+/+) or DNA-PKcs -/- HFFs were pre-treated with 2 µg/ml Nu7441 or carrier control before stimulation with htDNA or poly(I:C) for 8 hours and then immunoblotted with the indicated antibodies.

Supplementary Figure 3

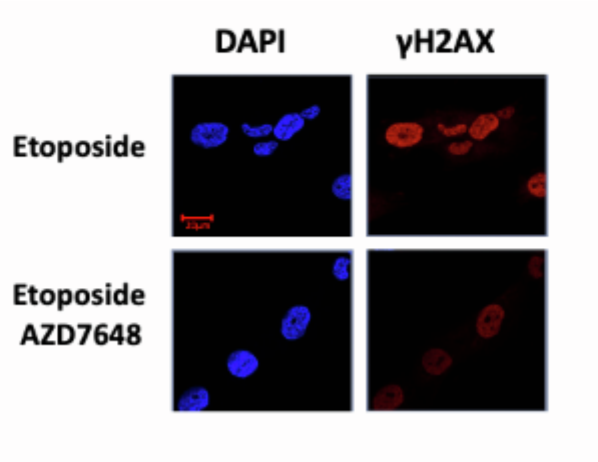

Supplementary Figure 3. **AZD7648 inhibits DNA-PKcs activity, related to Figure 3**. HFFs were pre-treated with 2 μM AZD7648 or carrier control and then with 30 μM etoposide for 2 h before being fixed and stained for anti-γH2AX. Scale bar = 20 μm

Supplementary Tables

**Plasmid name**	**sgRNA sequence**
PRKDC kinase sgRNA 1	GATCACGCCGCCAGTCTCCA
PRKDC kinase sgRNA 2	CAGACATCTGAACAACTTTA
TMEM173 (STING)	GGTGCCTGATAACCTGAGTA
MB21D1 (cGAS) sgRNA 1	CGGCCCCCATTCTCGTACGG
MB21D1 (cGAS) sgRNA 2	CGATGATATCTCCACGGCGG

Supplementary Table 1, related to STAR Method Details qRT-PCR: sgRNA primer sequences

**Gene**	**Forward primer**	**Reverse primer**
GAPDH	ACC CAG AAG ACT GTG GAT GG	TTC TAG ACG GCA GGT CAG GT
CXCL10	GTG GCA TTC AAG GAG TAC CTC	GCC TTC GAT TCT GGA TTC AGA CA
IFNB1	ACA TCC CTG AGG AGA TTA AGC A	GCC AGG AGG TTC TCA ACA ATA G
IFNL1	CGC CTT GGA AGA GTC ACT CA	GAA GCC TCA GGT CCC AAT TC
ISG54	CTG AAG AGT GCA GCT GCC TG	CAC TTT AAC CGT GTC CAC CC
NFKBIA	CTC CGA GAC TTT CGA GGA AAT	GCC ATT GTA GTT GGT AGC CTT

Supplementary Table 2, related to STAR Method Details, qRT-PCR: qRT-PCR primer sequences
